# Using Risk Models to Improve Patient Selection for High-Risk Vascular Surgery

**DOI:** 10.6064/2012/132370

**Published:** 2012-12-13

**Authors:** Philip P. Goodney

**Affiliations:** ^1^Section of Vascular Surgery, Dartmouth-Hitchcock Medical Center, 1 Medical Center Drive, Lebanon, NH 03766, USA; ^2^Dartmouth-Hitchcock Medical Center, The Dartmouth Institute for Health Policy and Clinical Practice, Hanover, NH 03765, USA

## Abstract

Vascular surgeons frequently perform procedures aimed at limiting death, stroke, or amputation on patients who present with diseases such as aortic aneurysms, carotid atherosclerosis, and peripheral arterial occlusive disease. However, now more than ever surgeons must balance the potential benefits associated with these interventions with the risks of physiologic insult for these elderly patients, who often have significant comorbidity burdens and the potential for costly complications. In this paper, we highlight how regional and national datasets can help surgeons identify which patients are most likely to benefit from vascular operations and which patients are most likely to suffer complications in the postoperative period. By using these guidelines to improve patient selection, our risk models can help patients, physicians, and policymakers improve the clinical effectiveness of surgical and endovascular treatments for vascular disease.

## 1. Introduction

Vascular surgeons frequently perform procedures aimed at limiting death, stroke, or amputation on patients who present with diseases such as aortic aneurysms, carotid atherosclerosis, and peripheral arterial occlusive disease [1]. These operations, whether they involve repair of an aneurysmal segment of aorta [2], bypass of an occluded artery in the leg [3], or removal of atherosclerotic plaque in a carotid artery [4], all serve a palliative purpose. The operations themselves, of course, do not cure atherosclerosis. Rather, they serve to mitigate its effects and limit its manifestations during the remainder of the patient's life. In other words, they must prevent death from ruptured aneurysms, limit limb loss from lower extremity vascular disease, and provide prophylaxis from stroke from carotid atherosclerosis.

 However, given the current cost constraints in health care, surgeons must, now more than ever, balance the potential benefits associated with these surgical interventions with alternative strategies [5, 6]. They must consider, against the background of medical alternatives, the physiologic insult that surgery entails in these elderly patients, who often carry with them significant comorbidity burdens [7–10]. One of the most important elements in effectively utilizing expensive, morbid procedures, such as thoracic aneurysm repair, lower extremity bypass, and carotid endarterectomy, is to perform these procedures on patients who will benefit from the procedure and not suffer complications from it. 

 While “good patient selection” may sound like a simple objective, achieving good patient selection remains a constantly evolving and sometimes elusive goal. New, less invasive endovascular alternatives can change the risk profile of an operation, such as thoracic endovascular grafting for thoracic aortic aneurysms [11]. New medications, such as high-dose statins and antiplatelet agents, can enhance the durability and effectiveness of lower extremity bypass procedures [12]. And both of these changes—less invasive alternatives and new medical adjuncts—have influenced which patients undergo, and garner benefit from carotid revascularization [13, 14]. 

 To best select patients for these high-risk operations, surgeons need data that incorporates these complex covariates in their decision-making algorithms. In this paper, we highlight how regional and national datasets can help surgeons identify which patients are most likely to benefit from these operations and which patients are most likely to suffer complications in the postoperative period. By using these guidelines to improve patient selection, our risk models can help patients, physicians, and policymakers improve the clinical effectiveness of surgical and endovascular treatments for vascular disease [15–18]. 

 Herein, we will use three illustrative examples—(1) survival following thoracic aneurysm repair [19], (2) amputation following lower extremity bypass [20], and (3) stroke following carotid revascularization [21]—to demonstrate how we can use past experience to inform which patients should be selected for vascular surgery in the future. 

## 2. Predicting Survival following Thoracic Aneurysm Repair

### 2.1. An Overview: What Is at Stake in Thoracic Aneurysm Repair?

 Given the significant potential for morbidity and mortality involved with descending thoracic aortic aneurysm (TAA) repair, surgeons have sought to balance the chance of dying from aneurysm rupture with the risk of surgical intervention [22–25]. Thoracic endovascular repair (TEVAR) has further complicated this relationship [26, 27], as this less-invasive intervention has expanded the pool of patients who are physiologically able to undergo surgery. Subsequently, single-center, regional, and national studies have described a significant increase of utilization of TEVAR, with a significant short-term benefit in perioperative mortality. 

 Several reports have described similar mid-term survival following open repair and TEVAR. However, these studies have been limited to single institution series from centers of excellence, or results from industry-sponsored trials and registries [23, 28], and only one clinical trial has reported five-year outcomes [26, 27]. Little population-based data describing TEVAR and open TAA repair is available that allows examination of survival differences for distinct TAA, especially across rupture status at presentation, or by repair type [28]. In this summary of our prior work, we describe our efforts in comparing perioperative and long-term mortality in the contemporary, real-world practice of open TAA repair and TEVAR. 

### 2.2. Establishing a Cohort of Patients Who Underwent Repair of TAA

**Figure 1:**
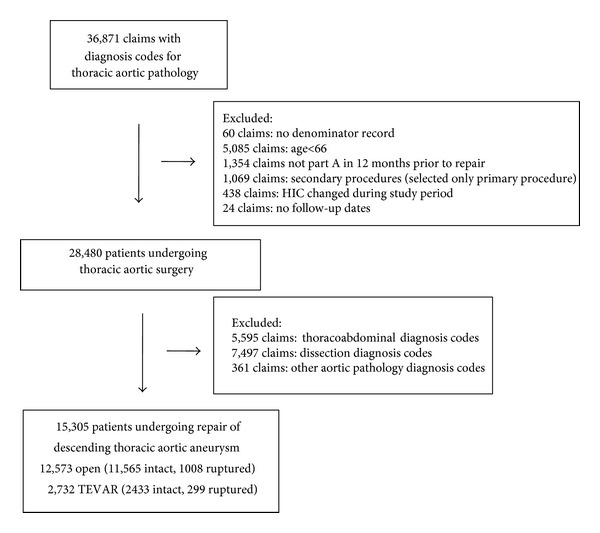
Establishing the cohort of patients undergoing TAA repair.

 First, using Medicare claims, we created a cohort of patients who underwent a broad range of thoracic aortic procedures, as defined by the ICD-9 codes [29]. Next, we selected from this cohort those patients who underwent TEVAR and open repair of TAA. As outlined in Figure 1, we eliminated all claims that were not contained in the Medicare Denominator File as well as patients not at least aged 66. We required patients to have at least one year of Medicare eligibility prior to surgery and used this time period to establish comorbidities and construct patient-specific Charlson scores [30, 31]. In addition to the procedural codes for TAA, we required that each patient has a diagnosis code for TAA. We excluded any patient with diagnosis codes for ascending thoracic aortic aneurysm, or with procedural codes for cardiopulmonary bypass occurring with circulatory arrest, and patients with thoracoabdominal aneurysms, thoracic aortic dissections, and “other” aortic pathology from our analysis, as these entities are clinically distinct from TAA. Finally, we examined the effect of changes in practice pattern over time. First, in prior work [32], we demonstrated that between 1998 and 2003, a significant increase in the use of TEVAR occurred. Before 2003, fewer than 10% of all intact TAA were repaired using TEVAR. After 2003, more than 10% of all intact TAAs were repaired with TEVAR, and this rate grew to 27% by 2007. We studied the effect of time across this pattern using time-dependent analyses. We followed this cohort over time to establish our two main outcome measures: perioperative and long-term survival, using one- and five-year survival rates. First, to define perioperative mortality, we sought to capture all deaths occurring in the period following surgery. We defined perioperative mortality as death occurring within the index hospitalization (regardless of postoperative day), as well as any death within thirty days (irrespective of inpatient or outpatient status). Second, survival at one and five years was established using the Medicare Denominator file to establish the date of death. We censored those patients who survived until the end of our analysis (at December 31, 2007). 

 Finally, we used a survivor function wherein age, gender, race, era of procedure, and Charlson score [30] were adjusted using a Cox proportional hazards model to estimate survival. This allowed comparison of survival estimates, adjusted to reflect survival within the strata of minimal patient-level risk within each group [33]. For ruptured patients, we stratified our Cox models by repair type and calculated hazard ratios for the remaining covariates. Additionally, we used propensity matching methods to create similar cohorts for survival analysis [34]. First, we generated a propensity score for the likelihood of undergoing TEVAR, based on a multivariable logistic model that described the association between preoperative patient characteristics and the choice to perform TEVAR. Within a sample of patients between age 65 and 75 who underwent surgery during the latter 4 years of our study period, we matched patients by age and comorbidity. This allowed us to generate two cohorts that were matched in terms of age, gender, race, era of procedure, comorbidities that constitute the Charlson score, as well as the Charlson score itself.

### 2.3. Comparison of Outcomes by Procedure Type

 In this analysis, we studied 12,573 Medicare patients who underwent open procedures and 2,732 patients who underwent TEVAR (Figure 1). By presentation status, 13,998 patients presented for surgery with intact TAA (11,565 open repair, 2,433 TEVAR), while 1,307 patients underwent surgery for ruptured TAA (1,008 open repair, 299 TEVAR). Several demographic differences existed between open repair and TEVAR patients with intact TAA. First, patients undergoing TEVAR were significantly older than patients undergoing open repair (75.9 years versus 73.8 years, *P* < 0.0001). Second, the proportion of male patients was slightly higher among patients undergoing TEVAR than open repair (58.7 versus 55.4%, *P* < 0.0001). 

**Figure 2:**
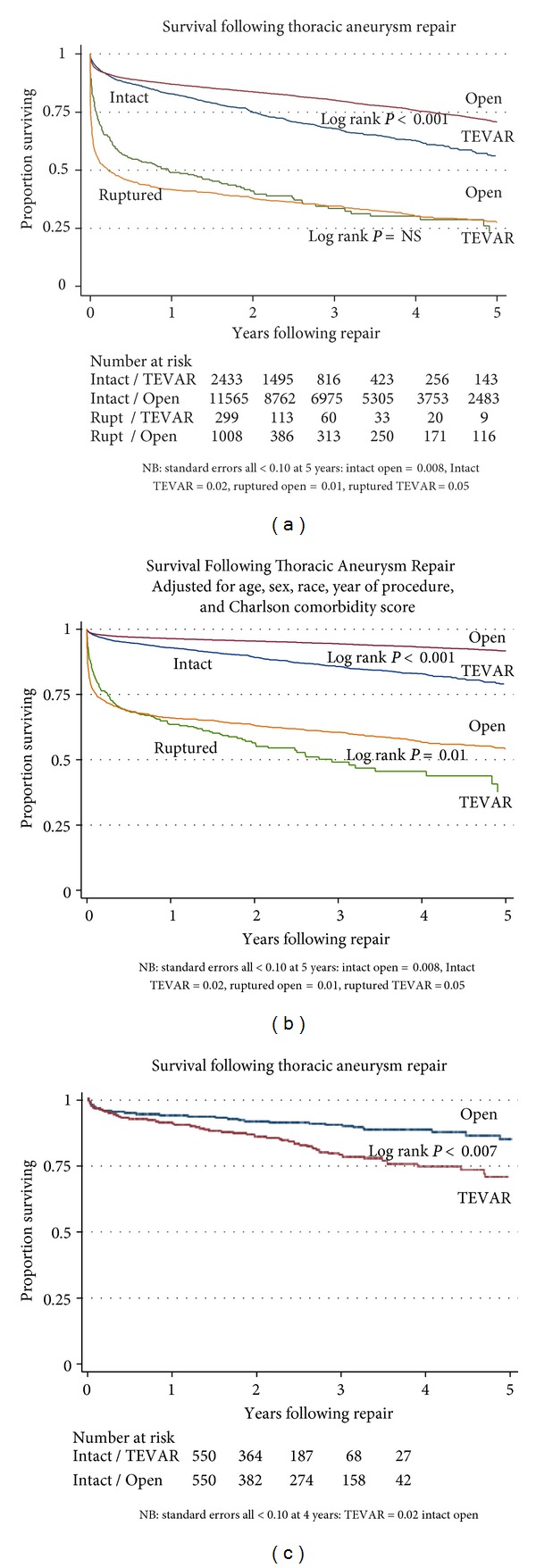
(a) Unadjusted five-year survival in thoracic aneurysms, by procedure type and diagnosis. (b) Adjusted five-year survival in thoracic aneurysms, by procedure type and diagnosis. Results represent male, nonblack patients under age of 75 with Charlson score < 2, performed after 2003. (c) Propensity-matched five-year survival in thoracic aneurysms, by procedure type. These patients represent a randomly selected, propensity-matched sample of low-risk patients who are at equal likelihood of undergoing either open repair or TEVAR.

 In terms of outcomes the lowest perioperative mortality rate occurred in patients undergoing repair of intact TAA using TEVAR (6.1% (95% CI 5.1–7.0%)). While the perioperative mortality rate for open repair was slightly higher (7.1% (95% CI 6.7–7.6%)), the clinical magnitude of this difference was small and borderline in terms of statistical significance (*P* = 0.07). Among patients presenting with ruptured thoracic aneurysms, perioperative mortality was 28.4% (95% CI 23.2–33.5%) for TEVAR and 45.6% (95% CI 42.5–48.7%) for open repair (*P* = 0.0001). Crude long-term survival varied by presentation (intact versus ruptured) as well as repair type (open repair versus TEVAR) is shown in Figure 2(a). Even though patients with intact TAA selected for TEVAR had lower perioperative mortality, patients selected for open repair reclaimed the survival advantage within the first year after surgery (1-year survival by life table analysis: 87% open repair (95% CI 86–88%), 82% TEVAR (95% CI 80–83%), log rank *P* = 0.001). This survival advantage continued to accumulate over time, as seen in our five-year survival data (5-year survival by life table analysis: 72% (95% CI 71–73%)) open repair, 62% TEVAR (95% CI 60–65%, log rank *P* = 0.001). This survival advantage was also seen in patients who underwent repair of ruptured TAA (Figure 2(b)). After five years, by life table analysis, fewer than 30% of patients were alive after repair of their ruptured TAA, irrespective of the type of repair (26% (95% CI 23–30%) open repair, 23% (95%CI 16–32%) TEVAR, log rank *P* = 0.37). Our results were similar, even in analyses adjusted for age, sex, race, era of procedure, and other comorbidities. 

In other adjusted analyses, we used propensity matching to create matched cohorts. These cohorts of patients were similar in terms of patient characteristics available for measurement in administrative claims and who underwent either open repair or TEVAR. In these groups, perioperative mortality was statistically similar in patients undergoing TEVAR compared to open repair (4.5% (95% CI 2.8–6.2%) open repair versus 4.2% (95% CI 2.5–5.8) TEVAR, *P* = 0.78). Any difference in perioperative mortality incurred by TEVAR disappeared within the first year postoperatively. Late survival was significantly worse in patients undergoing TEVAR (Figure 2(c)). By life table analysis at five years' postoperatively, late survival was significantly worse in patients undergoing TEVAR (73% (95% CI 68–76%)) than open repair (81% (95% CI 77–85%)) within the propensity-matched cohort (log rank *P* = 0.007). And lastly, our outcomes remained similar, even if we stratified by before or after FDA approval of TEVAR procedures. 

### 2.4. Implications of Our Findings, by Procedure Type

 The competing mortalities of observation and open surgical repair for TAA have been investigated for several decades. However, the effect of TEVAR on survival remains less well studied. Our study examined survival following open repair and TEVAR in national, real-world practice and found that, while perioperative mortality is lower in TEVAR, patients selected for TEVAR have worse long-term survival than patients selected for open repair. These results suggest that higher risk patients are being offered TEVAR and that some do not benefit based on long-term survival. This study demonstrates that the treatment of TAA follows a similar course to infrarenal AAA, with one important exception. As with infrarenal AAA, we found a survival advantage in short-term mortality for patients who undergo TEVAR as compared to open repair. Further, as with infrarenal AAA, any survival advantage gained in the perioperative period following endovascular repair was lost within two years after surgery. But unlike infrarenal AAA, adjusted survival at five years was significantly worse for patients selected for TEVAR as compared to open repair. Therefore, the widespread application of TEVAR has resulted in a cohort of patients who may have previously not undergone surgery, but now undergo TEVAR. Patients selected for TEVAR achieve worse survival than patients undergoing open repair, and many of these deaths occur within the first two years after TEVAR. These deaths could be due to the selection of “sicker” patients for TEVAR, although our finding of poorer survival following TEVAR persists, even in propensity-matched analyses that account for differences in patient risk measurable using administrative claims. Alternatively, these differences in survival could be explained by device-related complications occurring within the first five years following surgery.

 Survival following aortic aneurysm surgery has been a topic of extensive study, but primarily in patients with infrarenal abdominal aortic aneurysm (AAA) [35, 36]. Several randomized trials [37–39] have demonstrated lower perioperative mortality with endovascular techniques. It is well known that patients who experience late death following infrarenal AAA repair most commonly die from cardiopulmonary comorbidities unrelated to their aneurysm, and relatively few experience aneurysm-related death, in either open or endovascular repair [40]. As with patients with infrarenal AAA, we suspect that the loss of survival advantage is secondary to patient-level comorbidities. For example, in the EVAR-2 trial [41], survival was similar among patients treated with endovascular repair and patients who did not undergo repair. The data available from our study supports this presumption, as TEVAR patients tend to be older and have higher comorbidity scores than the patients selected for open repair. However, it is important to acknowledge that our findings are based solely on administrative claims, and our analysis is therefore limited in terms of clinical detail. 

 This study provides an important focus on the data used to demonstrate the efficacy of endovascular repair of TAA [42, 43]. First, when compared to data from TEVAR clinical trials (Table 1), it is evident that perioperative mortality is higher in “real-world” practice than in the centers of excellence where the clinical trials were performed. Second, in real-world practice, patients selected for open repair had better survival that the surgical controls used in clinical trials (72% five-year survival in Medicare, 67% five-year survival in the single trial that reported this measure). Collectively, these two differences resulted in the disparity in conclusions between our study, wherein TEVAR patients fared significantly worse at five years and the clinical trial, wherein outcomes were similar at five years. Given the findings in our study and others, there is little evidence to suggest that long-term survival is better in patients selected for TEVAR as compared to open surgical repair, and it may in fact be worse [44]. 

 In conclusion, the consistent short-term survival advantage offered by TEVAR disappears within the first two years after surgery. These results suggest that higher risk patients are being offered TEVAR and that some do not benefit based on long-term survival. Patient selection in TEVAR requires better risk stratification to better select patients for this new procedure. 

## 3. Lower Extremity Bypass: How Do We Select Patients Who Will Avoid Amputation?

### 3.1. Introduction

 Now, we turn our attention to a different type of vascular disease. Lower extremity peripheral arterial disease (PAD) affects over 8 million Americans, with significant associated morbidity and mortality [45–49]. Significant morbidity and mortality are encountered when elderly, medically complex patients undergo prolonged, invasive arterial reconstruction [47, 49]. One of the key elements in minimizing morbidity and mortality is optimal patient selection, especially in the current era wherein endovascular alternatives may exist [50]. To this end, we examined the risk factors associated with amputation or graft occlusion one year after surgery.

### 3.2. Studying a Cohort of Lower Extremity Bypass Patients Using a Regional Dataset

 In this analysis, we utilized data collected prospectively by the Vascular Study Group of Northern New England (VSGNNE), a regional cooperative quality improvement initiative developed in 2002 to study regional outcomes in vascular surgery. Further details on this registry have been published previously [51] and described in detail at vsgnne.org. Our unit of analysis was the bypass graft. Our main outcome measure was a combined measure of either permanent graft occlusion (loss of secondary patency) or major amputation (above or below knee) following surgery. Between January 1, 2003, and December 31, 2006, we identified 2,036 patients in our database that underwent 2,301 bypass procedures. Graft patency and major amputation rates were determined at several stages: first preoperatively, then at discharge following surgery, and again at 1 year postoperatively. Major amputation was defined as above-knee amputation or below-knee amputation. This outcome was assessed both at discharge and again at one-year followup. 

We used life table analysis given that not all follow-up data was obtained at exactly one year. Patients who died within one year of surgery were censored after their date of death. Risk factors found by univariate analysis to be associated with a *P* value of <0.1 were then used in a multivariate Cox proportional hazards model. This model was then used to calculate hazard ratios (HRs) and 95% confidence intervals (CIs) for ability to ambulate one year after surgery. To examine the validity of our risk prediction model, we constructed our risk model from the data obtained between 2003 and 2006. We then applied the risk model to the data acquired from the VSG sites in 2007. Lastly, using our model, we predicted a one-year amputation/occlusion rate for each patient, based on that patient's individual risk factor profile. We also compared results across centers, based on the characteristics of the patients treated at each center. The predictive ability of each model was then evaluated by generating an observed-to-expected outcome ratio across the range of risk identified. 

### 3.3. Outcomes and Their Association with Amputation

In our study, 2,036 patients underwent lower extremity bypass in Northern New England at one of the 11 centers participating in our registry. Patients were most commonly male (67%), aged 70–80 (30%), and nearly all were Caucasian. Nearly all patients had a history of either prior or current smoking (82%). About half of patients had a history of diabetes, 40% had coronary disease, and nearly a third had a history of COPD. Indication for surgery was claudication in 25% of patients, while the remaining patients had critical ischemia. In claudicants, one-year primary patency was 79% and secondary patency 87%. However, one-year primary and secondary patency was significantly lower (73 and 81%, respectfully) in patients with critical limb ischemia. As expected, limb salvage rates in our cohort were lower in patients with critical limb ischemia compared to claudication (86% versus 98%, *P* < 0.0001). Of 143 major amputations (8% of all bypasses) that occurred in our cohort, 17% were performed in patients who had a patent bypass graft. Similarly, 277 graft occlusions were noted in our cohort, of which 42% resulted in amputation. Our combined amputation or permanent graft occlusion rate was 3% at discharge and 16% at 1 year, by life table analysis. 

**Figure 3:**
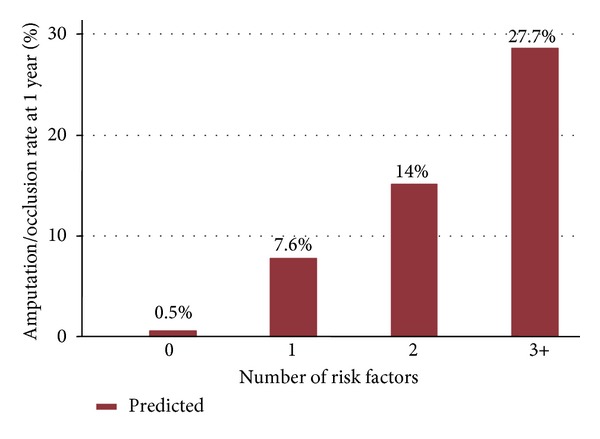
Predicted risk of amputation or graft occlusion, by the number of risk factors.

In univariate analyses, several physiologic parameters such as advanced age, diabetes, and hypertension were associated with amputation or graft occlusion at 1 year. Additionally, functional parameters such as baseline ambulatory status prior to surgery, living status prior to surgery, and presence of tissue loss were also highly closely correlated with loss of graft patency or amputation at 1 year. The factors associated with amputation or graft occlusion at one year are shown in Table 2. These were of age less than 50, nonambulatory status preoperatively, dialysis dependence, diabetes, critical limb ischemia, conduit requiring venovenostomy, tarsal target, and nursing home residence preoperatively. The likelihood of amputation or graft occlusion at one year varied from less than 0.5% in patients with no risk factors to nearly 30% in patients with three or more risk factors (Figure 3). 

**Figure 4:**
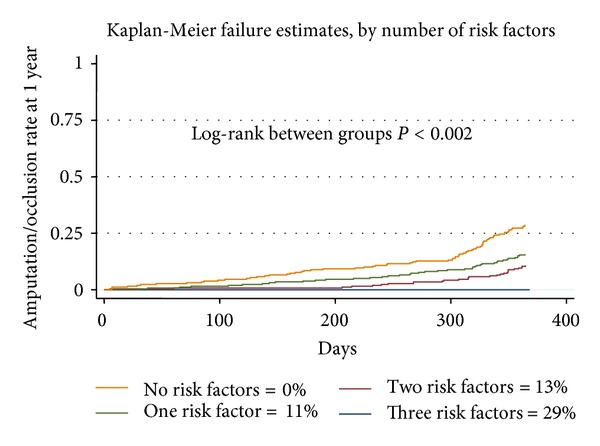
Validation of the risk prediction model using data from the VSGNE from 2007.

Lastly, to examine the validity of our risk prediction model, we constructed our risk model from the data obtained between 2003 and 2006 and applied the risk model to the data acquired from the VSG sites in 2007 (Figure 4). We found that our model performed well in discriminating risk. For example, patients with no risk factors had no episodes of graft occlusion or amputation, while those patients with three or more risk factors had a 30% incidence of amputation or graft occlusion. Differences between groups were statistically significant (<0.002). This risk prediction models allow benchmarking and facilitate comparison of risk-adjusted outcomes across centers as part of quality improvement efforts. To this end, we used our risk prediction model to calculate expected amputation/occlusion rates across centers and compared observed results with the expected results using O/E ratios. Predicted risks of amputation or graft occlusion varied from 3% at our lowest risk centers to 19% at the highest risk center. 

### 3.4. Implications of Our Findings for Patient Selection in Lower Extremity Bypass

 This review of factors associated with amputation or graft occlusion found that eight risk factors allow surgeons to predict which patients are at risk for graft occlusion or major amputation within one year following surgery. Additionally, this model allowed us to establish benchmarks for performance and compare risk-adjusted long-term outcomes within our region. 

 Prior investigators have recently studied clinical risk factors identified with a variety of different outcomes following lower extremity bypass [52–55]. In several single-center studies, ranging in size from 128 to 468 patients, investigators identified a variety of angiographic grading systems, as well as variables such as gender, conduit type, diabetes, and tissue loss, that were all predicted poor results in terms of graft patency. Further, Schanzer et al. analyzed a highly selected population of patients undergoing lower extremity bypass to determine predictors of amputation-free survival. They found that age, end-stage renal disease, critical limb ischemia, anemia, and advanced coronary artery disease were associated with decreased amputation-free survival one year after surgery. While these models are slightly different than the findings in our study, we believe both models will be helpful to both surgeons and patients in their preoperative discussions weighing the risks and benefits of surgery. 

 Our study, because it focuses on a predominantly white population, offers limited insight into racial effects on amputation risk. Peripheral vascular disease is more common in African Americans than any other racial group, and African American race has been widely identified as a risk factor for heart disease, stroke, and lower extremity vascular disease [56]. African Americans have poorer outcomes with lower extremity revascularization and overall are much more likely to undergo amputation [57–59]. In national studies, the Dartmouth Atlas Project has documented that African Americans underwent amputations at a rate nearly five times that of whites −4.17 per 1,000 African American Medicare patients, compared to 0.88 per 1,000 white Medicare patients [56]. Efforts to identify and address these disparities in care are ongoing and will be important to those surgeons caring for patients with vascular disease.

 Patients and surgeons can use this risk model not only to guide their expectations for success with bypass surgery but to also weigh the risks and benefits when considering surgery or endovascular intervention. Patients with only 1 or 2 risk factors have excellent outcomes in terms of graft patency and limb salvage, and in these patients bypass surgery represents a durable and effective treatment for both claudication and critical limb ischemia. However, in patients with 3 or more risk factors, nearly 1 in 3 will not have a patent graft or an intact limb within a year of their surgery. As to whether open bypass surgery or endovascular interventions should be used first, while many believe an “endovascular first” strategy should be employed [50, 60, 61], practice patterns vary significantly, and best practices have yet to be established with high quality evidence. We believe future comparative effectiveness research and clinical trials are necessary to address this question directly.

 But in addition to helping surgeons predict amputation risk, one of the main goals of the VSGNE is to study and improve the care of vascular surgery patients. By comparing risk adjusted outcomes, surgeons can set benchmarks and compare results across centers. By studying those centers that do things well, surgeons can identify processes of care that contribute towards the best outcomes. Then, by widely implementing these processes of care, we hope to improve our results across the region. This model has been used effectively in cardiac surgery [62, 63], myocardial infarction care [64, 65], and in the treatment of pulmonary disease [66] and cancer [67]. In addition to our ongoing efforts in quality improvement in lower extremity bypass surgery, we are applying similar strategies in improving results after carotid endarterectomy in our region [21]. 

 In conclusion, this study identified several risk factors that allow surgeons to predict which patients are at risk for amputation or graft occlusion following lower extremity bypass. Moreover, this model permits benchmarking and comparison of results across hospitals, key activities within our quality improvement initiative, which aims to use these data to broadly improve vascular care for patients at risk for amputation.

## 4. Carotid Atherosclerosis: Who Will Benefit from Carotid Revascularization?

### 4.1. Introduction: Who Is at Risk for Stroke during and after CEA?

Finally, we turn our attention to one last, and hotly debated, vascular territory—the carotid artery. While many believe that carotid endarterectomy, or surgical removal of plaque from the carotid arteries, can prevent stroke, debate has persisted for more than three decades as to which patients are best served by undergoing this procedure. Many investigators have studied risk factors associated with stroke or death following carotid endarterectomy (CEA), with the intent of improving preoperative assessment and patient selection [68–73]. Across these studies, the most consistent risk factor found to predict stroke or death following CEA has been preoperative neurologic symptoms (Table 3). Other variables associated with increased operative risk have included emergent operation [74], renal failure [75], and diabetes [72]. Interestingly, preoperative antiplatelet medications were found to be protective in only one of these models [76], despite evidence that such therapy reduces stroke risk associated with CEA [77, 78]. And, as with our efforts in lower extremity revascularization, another role for risk prediction models is to allow comparison of risk-adjusted outcomes among different centers. An important element in surgical quality improvement is to establish benchmarks for performance [79–82]. Most outcome benchmarks require risk adjustment, to account for potential differences in patient populations that could influence outcomes [83], and multivariate risk prediction models are developed to account for differences in patient characteristics when comparing outcomes [84, 85]. 

### 4.2. Selecting a Cohort of Carotid Endarterectomy Patients Using a Regional Dataset

As with the project described earlier, we included only patients in the Vascular Study Group of New England but in this analysis included those who underwent primary CEA (excluding redo operations or those combined with coronary bypass grafting). We selected 30-day postoperative stroke or death as our main outcome measure. The determination of major or minor stroke was made according to these definitions by the nurse clinician or trained research assistant responsible for entering the data into the database at each participating institution. Operative volume categories were defined using cutpoints guided by prior studies [86–88], modified to create terciles of approximately equal size from our dataset. In order to predict 30-day postoperative stroke or death, we initially performed univariate comparisons between our main outcome measures and many patient level variables. We selected appropriate patient preoperative variables for comparison based on previous publications [87, 89–92]. We generated a multivariate model, using backwards stepwise multivariate logistic regression, which was used to generate odds ratios and 95% confidence intervals for 30-day postoperative stroke or death. We used the resulting model to calculate an expected stroke/death rate for each patient, based on that particular patient's characteristics. 

### 4.3. Outcomes and Their Association with Stroke or Death

 Overall, we studied 2,714 patients undergoing 3,092 primary CEAs. Most patients were males, with a history of smoking and hypertension, and nearly half were symptomatic. We observed, across 3,092 CEAs, 38 minor strokes, 14 major strokes, and 8 deaths, 5 of which were stroke related, within 30 days of the index procedure (30-day stroke or death rate = 1.8%); 5 of the 52 strokes (10%) and 2 of the 8 deaths (25%) were reported after discharge but before 30 days. 

**Figure 5:**
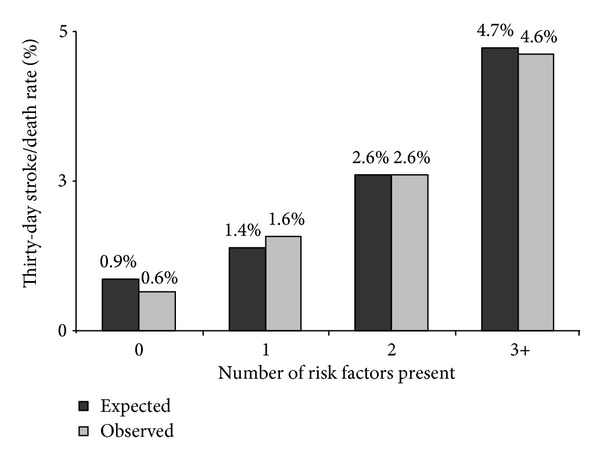
Comparison of observed and expected 30-day stroke/death rate. Risk factors include emergent procedure, preoperative ipsilateral stroke, age over 70, lack of antiplatelet agent, contralateral ICA occlusion, and congestive heart failure.

 We found that, on univariate analysis, emergent operation, contralateral internal carotid artery (ICA) occlusion, ipsilateral cortical symptoms (both TIA and stroke), congestive heart failure, performance of a completion study, and ICA stenosis <60% all were associated with increased risk of 30-day stroke or death. By multivariate analysis, emergent procedure, contralateral ICA occlusion, pre-operative ipsilateral cortical stroke, congestive heart failure, and age over 70 were associated with a significantly higher risk of stroke or death, while preoperative ASA or clopidogrel use was protective (Table 4). We examined the impact and additive effects of the number of risk factors present (Figure 5). In patients with 0-1 of these risk factors, the risk of stroke or death was less than 1%. However, when patients had 3 or more risk factors, the risk of stroke or death increased to nearly 5%. When expected stroke or death rates were compared to the observed stroke or death rates, excellent correlation was observed (*r* = 0.96). The model was found to have reasonable discriminative ability (area under ROC curve 0.71). Finally, in benchmarking analyses across the 8 hospitals in our region, the expected stroke/death rate ranged from 1.5% to 2.0%, while observed results varied from 0% to over 4%. Two hospitals had O/E ratios greater than 1, indicating a higher observed 30-day stroke and death rate than expected. 

### 4.4. Implications of Our Findings for Patient Selection in Carotid Endarterectomy

In our study of contemporary carotid surgical revascularization, we identified emergent procedure, contralateral ICA occlusion, preoperative cortical stroke, congestive heart failure, increased age, and (lack of) antiplatelet agent use as predictive of postoperative 30-day stroke or death following CEA. Our model describes several risk factors reported in previous studies, with similar effect sizes. Two prior models reported odds ratios of 30-day stroke or death of 1.8 for contralateral ICA occlusion, which is similar in magnitude to our odds ratio of 2.8 [18, 87]. These same studies [70, 72] also reported that ipsilateral cerebral symptoms increased the risk of stroke and death nearly twofold, similar to our finding of an odds ratio of 2.4 for preoperative ipsilateral stroke. Patients with pre-operative stroke before CEA have been shown to be at higher risk for postoperative stroke, independent of whether or not they have TIAs at the time of CEA [93]. And finally, emergent CEA, usually indicated for free-floating thrombus or crescendo symptoms, has been well described as having worse surgical outcomes compared to routine or urgent carotid endarterectomy [94], in keeping with our findings. 

Our study found a protective effect with antiplatelet therapy. The benefit of ASA to reduce postoperative stroke or death was shown in the North American Symptomatic Carotid Endarterectomy Trial [91], as well as a small randomized trial showed that stroke rates were reduced with lower dose perioperative ASA (81 mg/day) versus placebo [95]. Moreover, the ASA and Carotid Endarterectomy (ACE) trial [96] further emphasized the benefit of low-dose ASA. Finally, Kresowik et al. noted a significant reduction in stroke or death with the use of preoperative antiplatelet therapy in review of over 10,000 Medicare beneficiaries undergoing CEA between 1995 and 1996 [76]. Other antiplatelet agents have not been as extensively studied in terms of their benefit for stroke reduction after CEA, but recent studies of clopidogrel provide evidence that perioperative embolization is reduced [97, 98]. Given this evidence, one might assume that nearly all patients undergoing carotid revascularization are on antiplatelet medication. However, a recent study from The Netherlands indicated that only 66% of patients undergoing CEA were on preoperative antiplatelet agents, showing the opportunity for improvement using this simple process [77]. Even within our region, prior to starting our quality improvement efforts, only 73% of patients undergoing CEA were on antiplatelet agents. However, this percentage has increased to over 93% after dedicated efforts to improve this process measure across our participating centers [51]. We feel that our regional efforts in quality improvement have helped in this process, a technique that has been successfully used to improve processes of care by cardiac surgeons in our region [99]. 

We found it interesting that our study found no influence of surgeon or hospital volume on outcome; this is likely a type II error, given prior publications in this area [86–88]. Most studies that found a surgeon volume effect for CEA outcome required hundreds of surgeons and tens of thousands of patients to find a relatively small effect [86, 87], while we studied a relatively small number of surgeons performing approximately 3,000 CEAs. And finally, we found our stroke rates to be relatively low in our analysis, and we found little measurable benefit in statin therapy [100–102]. These findings may reflect a type II error, and future work will investigate these endpoints as our sample size increases. 

In conclusion, we identified factors associated with 30-day stroke or death following CEA using our regional prospective database. Surgeons can easily preoperatively “risk-stratify” patients by considering these easily available variables (emergent nature of procedure, contralateral ICA occlusion, preoperative ipsilateral cortical stroke, congestive heart failure, and age). From a quality improvement perspective, risk adjustment models like this allow valuable benchmarking among different centers and help regional collaboratives improve practice. 

## 5. Summary

 As we have demonstrated in these three examples, improving patient selection for high-risk vascular surgery involves a complex interaction between patient-level variables, procedure-related covariates, and insight the patient's underlying short- and long-term risk of adverse outcomes, all within the context of their life expectancy. As technology improves, analytic techniques evolve and our understanding of detailed regional and national datasets expands, we hope to eventually arrive at a time and place in vascular surgery where surgeons will know, *before* an intervention is performed, if their choice to perform surgery is the right one. And while a “crystal ball” that is able to predict surgical outcomes may seem like a far-fetched idea, it may not be as far off as you think.

## Figures and Tables

**Table 1:** Outcomes at 30 days, 1 year, and 5 years, by repair type and study.

Repair type	Outcome measure	Medicare 1998–2007	TAG [43]	VALOR [42]	TX2 [11]
	In hospital/30-day mortality	7.1%	6.4%	8.0%	6.0%
Open repair	1-year survival	87%	78%	79%	88%
	5-year survival	72%	67%	—	—

	In hospital/30-day mortality	6.1%	1.2%	2.0%	1.9%
TEVAR	1-year survival	82%	82%	84%	84–94%
	5-year survival	62%	68%	—	—

**Table 2:** Multivariate predictors of amputation or graft occlusion 1 year following lower extremity bypass.

Variable	Hazard ratio	95% CI	*P* value
Age			
**<40**	**1.4**	**0.4–4.7**	**0.645**
40–49	1.9	1.2–3.1	0.007
**50–59**	**1.2**	**0.8–1.7**	**0.424**
**60–69**	**1.2**	**0.8–1.7**	**0.334**
**70+**	**1**	**0.7–1.5**	**0.757**
Nonambulatory preoperatively	1.6	1–2.5	0.044
Dialysis	1.6	1.1–2.2	0.008
Diabetes	1.6	1.1–2.5	0.029
Critical limb ischemia	1.7	1.3–2.3	0.0001
Two vein segments	2	1.4–2.8	0.0001
Tarsal target for bypass	2.5	1.2–5.3	0.021
Nursing home residence	2.8	1.3–6	0.011

**Table 3:** Previous studies analyzing risk of stroke/death following CEA.

Author	Year	*n*, study type	Risk factors
Musser et al.	1994	562 patients, single center, retrospective series	AF, emergent operation, PVCs, intraop hypotension, ESRD
Goldstein et al.	1998	1,160 patients, multicenter, retrospective administrative database	Female, age over 75, CHF
Rothwell et al.	1999	2,060 patients, multicenter, prospective trial	Symptomatic status, DM, recent MI Plaque characteristics
Frawley et al.	2000	1,000 patients, single center retrospective series	Female gender
Kresowik et al.	2001	10,561 patients, retrospective chart review of medicare beneficiaries	Aspirin/ticlopidine use, heparin use, patch angioplasty
Tu et al.	2003	6,038 patients, regional retrospective database	Symptomatic status, AF, contralateral occlusion, CHF, DM
Nicolaides et al.	2005	1,115 patients, prospective clinical trial	Symptomatic status, degree of stenosis, creatinine

AF: atrial fibrillation; MI: myocardial infarction; PVC: premature ventricular contractions; ESRD: end stage renal disease; CHF: congestive heart failure; DM: diabetes mellitus.

**Table 4:** Multivariate analysis of factors associated with thirty-day stroke/death after CEA.

Variable	Odds ratio	95% CI	*P* value
Age over 70 years	1.3	0.8–2.3	0.315
Contralateral ICA occlusion	2.8	1.3–6.2	0.009
Antiplatelet agent use	0.4	0.2–0.9	0.02
Congestive heart failure	1.6	1.1–2.4	0.03
Emergent procedure (within 6 hours of admission)	7.0	1.8–26.9	0.004
Preoperative ipsilateral cortical symptoms (stroke)	2.4	1.1–5.1	0.02

Area under ROC: 0.71; TIA: transient ischemia attack.
